# Leukocyte poor platelet rich plasma vs leukocyte rich platelet rich plasma as a treatment for cervical facetogenic pain: A pooled analysis

**DOI:** 10.1016/j.inpm.2025.100566

**Published:** 2025-03-09

**Authors:** David J. Allison, Eldon Loh, Robert Burnham, Taylor Burnham, Ashley Smith

**Affiliations:** a Parkwood Institute, Lawson Health Research Institute, London, ON, Canada; b Department of Physical Medicine and Rehabilitation, Schulich Medicine and Dentistry, Western University, London, ON, Canada; c Department of Clinical Neurosciences, Cumming School of Medicine, University of Calgary, Calgary, Alberta, Canada; d VivoCura Health, #100, 325 Manning Rd NE, Calgary, Alberta, T2E 2P5, Canada; e Central Alberta Pain and Rehabilitation Institute, #1,6220 AB-2A, Lacombe, Alberta, T4L 2G5, Canada; f Division of Physical Medicine and Rehabilitation, University of Alberta, 116 St & 85 Ave, Edmonton, AB, T6G 2R3, Canada

**Keywords:** Cervical facetogenic pain, Platelet-rich plasma (PRP), Leukocyte-rich PRP (LR-PRP), Leukocyte-poor PRP (LP-PRP), Pain management, Neck disability index (NDI), Randomized controlled trial (RCT)

## Abstract

**Background:**

This study aimed to compare the effectiveness and safety of leukocyte-poor platelet-rich plasma (LP-PRP) and leukocyte-rich platelet-rich plasma (LR-PRP) for the treatment of cervical facetogenic pain through a pooled analysis of two independent studies. The goal was to provide preliminary evidence comparing the effect on pain relief and functional improvement over time to help inform future research.

**Methods:**

The pooled analysis integrated data from two studies: a prospective case series of LR-PRP (n = 36) and a randomized controlled trial (RCT) comparing LP-PRP (n = 21) with corticosteroid injections. Participants in both studies had chronic cervical facetogenic pain confirmed through medial branch blocks and were assessed at baseline, 3 months, and 6 months post-injection. Pain intensity was measured using the Numerical Pain Rating Scale (NRS), and functional disability was assessed using the Neck Disability Index (NDI). Linear mixed-effects models evaluated treatment effects over time, controlling for age, sex, and pain duration. Clinically important differences in pain and disability were determined. Adverse events were also recorded.

**Results:**

Both PRP groups exhibited significant improvements in pain and functional outcomes. However, LR-PRP demonstrated superior efficacy at 6 months, with a greater reduction in NRS scores (β = −1.68, p < 0.01, Cohen's d = −1.15) and NDI scores (β = −3.66, p = 0.02, Cohen's d = −0.94) compared to LP-PRP. A higher proportion of LR-PRP participants achieved clinically significant pain relief (≥2-point NRS reduction) and functional improvement (≥10 % NDI reduction) at 6-months. No significant differences were observed at 3 months. Adverse events were more common in the LP-PRP group, with 11 documented cases, including increased pain and muscle spasms, whereas the LR-PRP group reported no adverse events.

**Conclusion:**

These preliminary findings suggest that LR-PRP appears to provide greater long-term benefits for cervical facetogenic pain compared to LP-PRP, with superior pain reduction, improved function, and a favorable safety profile. These findings suggest the higher leukocyte concentration in LR-PRP may enhance therapeutic outcomes for cervical pain. Further large-scale, randomized trials are necessary to confirm these results and establish standardized PRP preparation protocols.

## Highlights

• LR-PRP may offer more long-term benefits than LP-PRP for cervical facetogenic pain.
• Preliminary evidence for superior pain, function, and safety outcomes for LR-PRP.
• These findings suggest LR-PRP may be superior and warrant future RCT confirmation.

## Abbreviations

LOCF: Last Observation Carried Forward;
LP-PRP: Leukocyte-Poor Platelet-Rich Plasma
LR-PRP: Leukocyte-Rich Platelet-Rich Plasma
MBB: Medial Branch Block
MCID: Minimal Clinically Important Difference
NDI: Neck Disability Index
NRS: Numerical Pain Rating Scale
OA: Osteoarthritis
PRP: Platelet-Rich Plasma
RCT: Randomized Controlled Trial
SE: Standard Error
SD: Standard Deviation
CI: Confidence Interval
ITT: Intention-to-Treat

## 1 Introduction

Platelet-rich plasma (PRP) is increasingly recognized as a promising orthobiological treatment for musculoskeletal conditions and has recently garnered attention for its potential to treat cervical facetogenic pain. There remain questions regarding the advantages of different PRP formulations, particularly between leukocyte-rich PRP (LR-PRP) and leukocyte-poor PRP (LP-PRP). LR-PRP contains a higher leukocyte concentration, which may enhance the immune response and facilitate tissue repair [1]. This elevated leukocyte count may also increase inflammation, potentially hindering the healing process and exacerbating pain [[2], [3], [4]]. However, as inflammation is also a necessary phase of the healing process, leukocytes may play an important role in the process of “inflammatory regeneration” [5]. In contrast, the reduced leukocyte content of LP-PRP may minimize inflammation while still utilizing platelets to release essential growth factors such as platelet-derived growth factor and vascular endothelial growth factor that are critical for tissue regeneration [1]. Thus, while PRP has emerged as a potential therapeutic option for various musculoskeletal conditions, including cervical facetogenic pain, it is currently unclear which preparation is optimal [[6], [7], [8]].

While a comparison of these different preparations in patients with cervical facetogenic pain has not been performed, a meta-analysis of 32 studies on knee osteoarthritis (OA) offers some insight, suggesting that both formulations may be equally effective in terms of pain relief and functional improvement. LP-PRP was shown to be associated with fewer adverse reactions, including post-injection pain and swelling, highlighting a potential safety advantage [9]. Notably, this meta-analysis included only one prospective study which directly compared LP-PRP and LR-PRP, underscoring the need for further research in this area.

Our research team previously conducted a prospective case series that demonstrated the effectiveness of LR-PRP in treating cervical facetogenic pain [10]. More recently, we completed a randomized controlled trial (RCT) comparing intra-articular LP-PRP injections with corticosteroid injections into the cervical facets, showing similar benefit between LP-PRP and corticosteroids [11]. However, no study has yet directly compared LP-PRP and LR-PRP in this clinical context.

This pooled analysis seeks to address this gap by comparing the effectiveness of LP-PRP and LR-PRP for the treatment of cervical facetogenic pain. By doing so, this study will provide a preliminary assessment on the comparative effectiveness and safety of these two PRP formulations, helping to inform future trials and potentially lead to additional treatment strategies for cervical facetogenic pain.

## 2 Materials and methods

### 2.1 Study design

This pooled analysis integrates data from two separate studies investigating treatments for cervical facetogenic pain. Detailed methods for each of these studies has been previously published [11,12]. In brief, the first study was a prospective case series, involving 36 participants who received a total of 2 ml of intra-articular leukocyte-rich PRP (LR-PRP). The second was a randomized controlled trial (RCT), involving 40 participants who were randomized to receive either a 1 ml intra-articular injection of leukocyte-poor PRP (LP-PRP) (n = 21) or corticosteroid injections (n = 19). Participants who received LP-PRP have been included in the current analysis. Both studies followed patients longitudinally, with assessments at multiple time points, including baseline and post-treatment follow-ups at 3-, and 6-months. All data used in this analysis were derived from studies approved by their respective institutional review boards.

### 2.2 Participants

Participants in the prospective case series were individuals with chronic cervical facetogenic pain, confirmed via single diagnostic medial branch blocks (MBBs), using 0.3 ml of local anesthetic per medial branch, with at least 50 % pain relief. The RCT recruited participants who met similar criteria, with chronic cervical pain confirmed by dual MBBs (using 0.5 ml of local anesthetic per medial branch) and a Numerical Pain Rating (NRS) score ≥4/10. Exclusion criteria for both studies included 1) Known or suspected serious spinal pathology (e.g. metastatic disease of the spine), 2) Confirmed cervical fracture or dislocation at time of injury, 3) Spinal surgery or radiofrequency neurotomy of the cervical spine in the past 12 months, unless pain had returned for at least 1 month prior to participation in the study, 4) cervical facet IA corticosteroid injection in the past four months, unless pain had returned to baseline for at least one month, 5) History of any uncontrolled mental health conditions, 6) Other contraindications to spinal injection (e.g bleeding diathesis or inability to discontinue anticoagulants). Across both studies, a total of 57 participants were included in the pooled analysis.

### 2.3 Study Interventions

In the case series, all participants received LR-PRP prepared using an in-house protocol designed to enhance leukocyte concentration [10], The PRP was prepared by drawing 13 mL of whole blood into a 20 mL sterile syringe that had been preloaded with 2 mL of 4 % sodium citrate. The syringe was then capped, modified by trimming the plunger and tip, and placed into a 50 mL conical tube with the capped end facing upward. It was subsequently centrifuged at 1500 G for 7 min to separate the blood into an upper plasma layer, a middle buffy coat, and a lower red blood cell layer. The upper portion of the plasma was discarded, and the bottom 3.5 mL, comprising the buffy coat along with 0.5 mL of the top red cell layer, was drawn into a separate syringe for injection. In-house quality assurance testing had confirmed that the resulting PRP had a platelet concentration approximately 4.2 times that of whole blood, while neutrophils, lymphocytes/monocytes, and red blood cells remained near baseline levels [12]. In the RCT, participants were randomized to receive either LP-PRP or corticosteroid injections. PRP in the RCT was prepared using standard protocol of the Arthrex ACP Double-Syringe System for Autologous Conditioned Plasma (Arthrex, Inc.; Naples, Florida, USA), which provides a LP-PRP formulation based on lab analyses [13]. Injections in both studies were performed at the same levels as prior successful cervical MBB. LR-PRP injection in the case series was done under fluoroscopic ± ultrasound guidance, while LP-PRP injection in the RCT used fluoroscopic guidance.

## 3 Outcomes

The primary outcome for both studies was cervical facetogenic pain intensity (NRS). Secondary outcomes included functional disability, assessed by raw scores (0–50) on the Neck Disability Index (NDI), and clinically important differences. Clinically important differences in NRS scores were based on the proportion of participants achieving ≥50 %, as well as ≥2-point improvement. Clinically important differences in NDI scores were based on the proportion of participants achieving at least a 10 % improvement [14]. Treatment-emergent adverse events as previously defined [10,11] were also compared between groups.

### 3.1 Statistical analysis

Overall summary statistics are presented as mean ± SD for continuous variables and frequencies or percentages for discrete variables. The analysis was performed as an intention-to-treat (ITT) analysis with last observation carried forward (LOCF) for missing data as well as a per protocol analysis including only participants with complete datasets. Participants with no data at any timepoint were excluded from analyses.

For NRS and NDI scores, linear mixed effect models were used to assess the impact of treatment (leukocyte poor [LP] PRP and leukocyte rich [LR] PRP) over time (baseline, 3-months post, 6-months post). Age, sex, and pain duration were entered as co-variates and kept in the model if significant. The model included fixed effects for treatment group, time, and their interaction, as well as the covariates age, sex, and pain duration. Random intercepts were included for subjects to account for individual differences in baseline scores. Residuals were assessed for normality through Shapiro-Wilk tests and QQ-plots. To further evaluate for clinically important differences, Chi squared analysis was performed to evaluate the relationship between treatment condition and the proportion of patients achieving a 50 %, as well as 2-point improvement in NRS scores of pain intensity (calculated with 95 % confidence intervals [95 % CI]) at 3- and 6-months. Additionally, clinically important differences for NDI scores are presented based on the proportion of participants achieving a 10 % improvement [14]. Statistical significance was set at p < 0.05 for all tests. Effect sizes were calculated for both time and interaction effects and expressed as Cohen's d. Categorically, values were considered ‘small’ between 0.2 or 0.3; ‘medium’ for values exceeding 0.3, but less than 0.8 and ‘large’ for values greater than 0.8 [15]. Statistical analyses were performed using R (version R-4.3.1).

## 4 RESULTS

### 4.1 Data availability for both the LP- and LR-PRP studies are presented in Table 1

Demographic characteristics of included participants are shown in Table 2. There was a significant between group difference for age (t(55) = 2.18, p = 0.03) with the LP group being significantly older than the LR group. There was also a trend towards group differences for sex (x^2^ = 3.15, df = 1, p < 0.08), with the LR group showing a higher proportion of females. Last, there was a strong trend (t(55) = −1.98, p = 0.053) for a between group difference in pain duration with the LP group showing a shorter duration of pain.

**Table 1:** Data Availability.

Variable	LP-PRP RCT (n = 21)	LR-PRP Case Study (n = 36)
***Screened***	89	44
***Eligible***	40	36
***Randomized***	21 (to PRP treatment)	**--**
***Baseline Data***	All data available	NRS: 36, NDI: 31
***3-Month Data***	All data available	NRS: 35, NDI: 30
***6-Month Data***	All data available	NRS: 24, NDI: 23

**Table 2:** Participant Demographics.

Sex	Total [n (%)]	LP [n (%)]	LR [n (%)]
Male	13 (22.8 %)	8 (38.1 %)	5 (13.9 %)
Female	44 (77.2 %)	13 (61.9 %)	31 (86.1 %)

	**Total [Mean ± SD (n)]**	**LP [Mean ± SD (n)]**	**LR [Mean ± SD (n)]**
**Age (years)**	48.5 ± 11.9 (57)	52.9 ± 12.4 (21)	46.0 ± 10.9 (36)
**Pain Duration (months)**	26.8 ± 29.4 (57)	17.0 ± 13.3 (21)	32.6 ± 34.6 (36)

### 4.2 NRS – cervical facetogenic pain intensity

Participants from both the LP- and LR-PRP studies presented with moderate to severe levels of pain at baseline [16]. The intention to treat (ITT) analysis showed no significant baseline differences in neck pain intensity between LP- and LR-treatment groups (p = 0.34). A significant group∗time interaction at 6-months (β = −1.68, SE = 0.57, 95 % CI = −2.78, −0.57, t = −2.95, p < 0.01, Cohen's d = −1.15) suggests the LR-group had a greater improvement from baseline to 6-months compared to the LP- group. Similar results were found using a per protocol analysis. No significant baseline between group differences were shown (p = 0.40). There was a significant group∗time interaction at 6-months (β = −1.55, SE = 0.61, 95 % CI = −2.74, −0.37, t = −2.54, p = 0.01, Cohen's d = −1.07) (See Table 3).

**Table 3:** NRS scores of pain intensity.

NRS – Cervical Facetogenic Pain Intensity				
	*ITT*		*Per Protocol*	
***Time***	***LP****[Mean ± SD (n)]*	***LR****[Mean ± SD (n)]*	***LP****[Mean ± SD (n)]*	***LR****[Mean ± SD (n)]*
***Baseline***	6.24 ± 1.81 (21)	6.39 ± 1.59 (36)	6.24 ± 1.81 (21)	6.67 ± 1.61 (24)
***3 Month***	4.81 ± 2.77 (21)	4.08 ± 2.31 (36)	4.81 ± 2.77 (21)	4.46 ± 2.41 (24)
***6 Month***	5.67 ± 2.01 (21)	4.14 ± 2.21 (36)	5.67 ± 2.01 (21)	4.54 ± 2.25 (24)

With respect to clinically important differences, a significantly higher proportion of patients of the LR group achieved a ≥2-point reduction in NRS scores at 6-months post-injection (both in the ITT and per protocol analysis). No significant differences in the proportion of participants achieving 50 % pain relief or a ≥2-point reduction in NRS scores were found between LP- and LR-groups at 3-months post-injection (See Table 4).

**Table 4:** Clinically relevant differences for NRS scores of pain intensity.

50 % Pain Relief - ITT						
	Leukocyte Poor		Leukocyte Rich			
*Time*	n/Total	Proportion (95 % CI)	n/Total	Proportion (95 % CI)	χ^2^	p
**3 Month**	6/21	28.6 (12.2–52.3)	15/36	41.7 (26.0–59.1)	0.50	0.48
**6 Month**	3/21	14.4 (3.8–37.4)	14/36	38.9 (23.6–56.5)	2.75	0.10

***50 % Pain Relief – Per Protocol***						
	**Leukocyte Poor**		**Leukocyte Rich**			
***Time***	**n/Total**	**Proportion (95 % CI)**	**n/Total**	**Proportion (95 % CI)**	**χ**^**2**^	**p**
**3 Month**	6/21	28.6 (12.2–52.3)	8/24	33.3 (16.4–55.3)	<0.01	0.98
**6 Month**	3/21	14.4 (3.8–37.4)	7/24	29.2 (13.4–51.2)	0.70	0.40

**MCID (>2 point NRS reduction) - ITT**						
	**Leukocyte Poor**		**Leukocyte Rich**			
**Time**	**n/Total**	**Proportion (95 % CI)**	**n/Total**	**Proportion (95 % CI)**	**χ**^**2**^	**p**
**3 Month**	12/21	57.1 (34.4–77.4)	22/36	61.1 (43.5–76.4)	<0.01	0.99
**6 Month**	6/21	28.6 (12.2–52.3)	23/36	63.9 (46.2–78.7)	5.28	0.02

**MCID (>2 point NRS reduction) – Per Protocol**						
	**Leukocyte Poor**		**Leukocyte Rich**			
**Time**	**n/Total**	**Proportion (95 % CI)**	**n/Total**	**Proportion (95 % CI)**	**χ**^**2**^	**p**
**3 Month**	12/21	57.1 (34.4–77.4)	15/24	62.5 (40.8–80.4)	<0.01	0.95
**6 Month**	6/21	28.6 (12.2–52.3)	16/24	66.7 (44.7–83.6)	5.07	0.02

### 4.3 NDI – self rated disability

Participants from both the LP- and LR-PRP studies presented with moderate levels of disability at baseline [17]. The ITT analysis found no significant baseline differences in self-rated disability between LP and LR treatment groups (p = 0.70). A significant group∗time interaction at 6-months (β = −3.66, SE = 1.56, 95 % CI = −6.68, −0.64, t = −2.35, p = 0.02, Cohen's d = −0.94) suggests the LR group had a greater improvement in self-rated disability from baseline to 6-months compared to the LP group. Similar results were found using a per protocol analysis. No significant baseline between group differences were shown (p = 0.78). There was a significant group∗time interaction at 6-months (β = −3.50, SE = 1.71, 95 % CI = −6.80, −0.20, t = −2.05, p = 0.04, Cohen's d = −0.88) (See Table 5).

**Table 5:** NDI scores of self-rated disability.

NDI – Self Rate Disability				
	*ITT*		*Per Protocol*	
***Time***	***LP****[Mean ± SD (n)]*	***LR****[Mean ± SD (n)]*	***LP****[Mean ± SD (n)]*	***LR****[Mean ± SD (n)]*
***Baseline***	22.0 ± 6.88 (21)	23.2 ± 6.42 (31)	22.0 ± 6.88 (21)	23.4 ± 7.06 (23)
***3 Month***	17.6 ± 8.82 (21)	16.3 ± 6.25 (31)	17.6 ± 8.82 (21)	16.9 ± 6.66 (23)
***6 Month***	18.2 ± 8.68 (21)	15.8 ± 5.59 (31)	18.2 ± 8.68 (21)	16.2 ± 5.87 (23)

With respect to clinically important differences, a significantly higher proportion of patients of the LR group achieved a ≥10 % improvement in NDI score at 6-months post-injection (both in the ITT and per protocol analysis). No significant difference in the proportion of participants achieving a 10 % improvement in NDI score was found between LP- and LR-groups at 3-months post-injection (See Table 6).

**Table 6:** Clinically relevant differences for NDI scores of self-rated disability.

MCID (10 % NDI reduction) - ITT						
	Leukocyte Poor		Leukocyte Rich			
Time	n/Total	Proportion (95 % CI)	n/Total	Proportion (95 % CI)	χ^2^	p
**3 Month**	13/21	61.9 (38.7–81.0)	26/31	83.9 (65.5–93.9)	2.16	0.14
**6 Month**	13/21	61.9 (38.7–81.0)	28/31	90.3 (73.1–97.5)	4.48	0.03

**MCID (10 % NDI reduction) – Per Protocol**						
	**Leukocyte Poor**		**Leukocyte Rich**			
**Time**	**n/Total**	**Proportion (95 % CI)**	**n/Total**	**Proportion (95 % CI)**	**χ**^**2**^	**p**
**3 Month**	13/21	61.9 (38.7–81.0)	19/23	82.6 (60.5–94.3)	1.44	0.23
**6 Month**	13/21	61.9 (38.7–81.0)	21/23	91.3 (70.5–98.5)	3.86	0.04

### 4.4 Adverse events

In the LP-PRP group, a total of 11 adverse events were documented. These included tingling sensations from the neck to the shoulder (n = 1), increased cervical facetogenic pain (n = 3), back pain (n = 1), shoulder pain (n = 1), arthritis flare-ups (n = 1), spasms in the lower neck (n = 1), spasms in the shoulder (n = 1), and headache (n = 2). No adverse events were reported in the LR-PRP group.

## 5 Discussion

Both LP-PRP and LR-PRP groups demonstrated improvements in cervical facetogenic pain intensity and self-rated disability at 3- and 6-months post-injection. However, at 6 months, the LR-PRP group showed superior outcomes, with significantly greater reductions in pain intensity and disability. A larger proportion of participants in the LR-PRP group also achieved clinically relevant improvements, including ≥2 points reductions in NRS scores and ≥10 % improvement in NDI scores. These findings suggest that LR-PRP may offer enhanced benefits for cervical facetogenic pain over LP-PRP, particularly in the longer term.

Previous studies have not directly compared LP-PRP and LR-PRP for the treatment of cervical facetogenic pain, although research in other musculoskeletal conditions, such as knee osteoarthritis (OA), provides some context. A study by Filardo et al. (2012) involving patients with knee OA found that both PRP formulations improved pain and function, but LR-PRP was associated with higher rates of post-injection pain and swelling [18]. These results align with a meta-analysis by Kim et al. (2021), which suggested that while both PRP types are similarly effective for OA, LP-PRP has a better safety profile due to its lower incidence of adverse reactions, such as inflammation and pain [9]. Further, a meta-analysis by Abbas et al. (2022) found no statistically significant or clinically meaningful differences in any patient outcomes between LP-PRP and LR-PRP [19].

Our findings contrast with earlier studies in knee osteoarthritis (OA), as LR-PRP demonstrated superior effectiveness in reducing cervical facetogenic pain and disability without an accompanying increase in adverse events. The reasons for this difference in outcomes at different joints are not clear. However, the cervical facet population included those with whiplash-associated disorders, and therefore likely capsular strain/sprain [20]. The presence of capsular sprain/strain, as opposed to the chronic intra-articular joint changes that may be present in knee OA may account, in part, for the difference in outcomes.

One factor contributing to the variability in outcomes when comparing the efficacy of LP-PRP and LR-PRP is the lack of standardized PRP preparation protocols. Differences in centrifugation methods can lead to variable platelet and leukocyte concentrations, resulting in significant heterogeneity in PRP formulations across studies [21]. For example, in the Filardo et al. (2012) study, the LP-PRP preparation had substantially lower platelet concentrations compared to the LR-PRP preparation. Our study may have been similarly limited, as while the LR-PRP preparation was shown to have a platelet concentration of approximately 4.2x that of whole blood, the LP-PRP (prepared using the Arthrex ACP device) was not routinely quantified for its platelet count. For a robust efficacy comparison, ideally both PRP preparations would have similar platelet counts; without these data, it is unclear whether the differences in clinical outcomes are solely attributable to variations in leukocyte concentration or if they are also influenced by differences in platelet counts. Future studies should incorporate systematic, quantitative analyses of all cellular components to more clearly delineate the relationship between specific PRP characteristics and clinical efficacy.

Additional important limitations to the current analysis should be acknowledged. The primary limitation for this study is the use of a pooled analysis which combined data from a prospective case series on LR-PRP and a randomized controlled trial on LP-PRP. This approach introduces bias due to differences in study design, patient selection, control mechanisms, and blinding practices. Additionally, the use of different PRP preparation methods and injection techniques, along with variability in inclusion criteria, further limits direct comparability between the two groups.

Despite the noted limitations, this study provides the first comparative analysis of LP-PRP and LR-PRP for the treatment of cervical facetogenic pain. Our study has several strengths, including the use of rigorous statistical models and the inclusion of both intention-to-treat and per-protocol analyses to ensure robust findings. The assessment of both pain intensity and functional disability over a 6-month follow-up period provides a comprehensive evaluation of PRP effectiveness in this clinical context. Additionally, we statistically controlled for age, sex, and pain duration given the potential influence on PRP efficacy as demonstrated in previous trials [18]. Thus, while this study offers valuable preliminary insights into the effectiveness of LP-PRP and LR-PRP for the treatment of cervical facetogenic pain, it is a hypothesis-generating analysis that is exploratory in nature and not intended to provide definitive evidence regarding the superiority of one treatment over the other. Future larger scale, blinded RCT's, with longer follow-up periods are needed. The development of standardized PRP preparation protocols will also be important to allow for more reliable comparisons of LP-PRP and LR-PRP efficacy across studies.

## 6 Conclusion

This pooled analysis demonstrates that both leukocyte-poor platelet-rich plasma (LP-PRP) and leukocyte-rich platelet-rich plasma (LR-PRP) effectively reduce cervical facetogenic pain and improve functional disability. Notably, our findings indicate that LR-PRP might offer superior outcomes at the six-month follow-up, achieving greater reductions in pain intensity and disability scores compared to LP-PRP. While these findings suggest that the higher leukocyte concentration may enhance therapeutic efficacy for cervical facetogenic pain, distinguishing it from previous findings in knee osteoarthritis, the limitations of combining data from studies with differing designs, patient populations, and protocols require cautious interpretation. These exploratory findings serve as a basis for generating more specific hypotheses, and future large-scale, blinded randomized controlled trials with standardized PRP preparation methods and extended follow-up periods are necessary to validate and refine these observations.

## Funding

This study was funded by the 10.13039/100010564Academic Medical Organization of Southwestern Ontario (AMOSO)

## Declaration of competing interest

The authors declare the following financial interests/personal relationships which may be considered as potential competing interests:Eldon Loh reports financial support was provided by Academic Medical Organization of Southwestern Ontario. If there are other authors, they declare that they have no known competing financial interests or personal relationships that could have appeared to influence the work reported in this paper.
